# Benchmarking of commercially available CHO cell culture media for antibody production

**DOI:** 10.1186/1753-6561-7-S6-P13

**Published:** 2013-12-04

**Authors:** David Reinhart, Christian Kaisermayer, Lukas Damjanovic, Renate Kunert

**Affiliations:** 1 Dept. of Biotechnology, University of Natural Resources and Life Sciences, Muthgasse 11, 1190 Vienna, Austria; 2 GE Healthcare Life Sciences AB, Björkgatan 30, 75184 Uppsala, Sweden

## Introduction

Chinese hamster ovary (CHO) cells have become the preferred expression system for the production of complex recombinant proteins. Several suppliers offer CHO specific cell cultivation media and sometimes also media systems, which combine feeds and basal medium. We compared eight commercially available CHO cell culture media and feed supplements from three different vendors to evaluate their influence on cell growth and antibody production of a CHO cell line. In conclusion, ActiCHO™ Media System, with a matching base media and feeds, resulted in the highest cell growth and the highest productivity. Further nutrient additions did not have a profound effect on the process performance.

## Materials and methods

Cultivation media:

ActiCHO P (GE Healthcare)

CD CHO (Life Technologies)

CD OptiCHO™ (Life Technologies)

CD FortiCHO™ (Life Technologies)

Ex-Cell™ CD CHO (Sigma Aldrich)

ProCHO 5 (Lonza)

BalanCD™ CHO Growth A (Irvine Scientific)

Cellvento™ CHO-100 (EMD Millipore)

• Anti-Clumping Agent (Life Technologies)

• CHO DG44 cells expressing an IgG antibody

• Cultivation conditions: 37°C, 7% CO_2_, 140 rpm

• Batch and fed-batch cultivations were run in Erlenmeyer shake flasks (Corning, NY). The cultures were grown in a CO_2 _incubator shaker (Kühner, Switzerland)

• Batch cultures were run as single experiments, the method variability was determined by a triplicate reference experiment in ActiCHO P.

• During fed-batch processes the cultures were fed with the corresponding feeds ActiCHO Feed A and Feed B (GE Healthcare), BalanCD™ CHO Feed 1 (Irvine Scientific) or EfficientFeed™ A and/or FunctionMAX™ (both Life Technologies) according to the manufacturers inctructions [1]. The respective feeding regimens are shown in Table 1.

**Table 1:** Feeding regimens in fed-batch cultures.

Basal medium	ActiCHO Feed A	ActiCHO Feed B	EfficientFeed A	FunctionMAX	Feed 1	Peak cell conc. [10^6 ^c/ml]	Harvest Titer [g/L]
ActiCHO P	daily; 3%	daily; 0.3%	-	-		23.9	5.48

ActiCHO P	daily; 3%	daily; 0.3%	-	3, 5, 7; 3.3%		21.3	5.82

CD OptiCHO	-	-	3, 5, 7, 9; 10%	-		5.8	0.72

CD OptiCHO	-	-	3, 5, 7; 10%	-		5.2	0.80

CD OptiCHO	-	-	3, 5, 7; 10%	3, 5, 7; 3.3%		6.3	1.74

CD OptiCHO	daily; 3%	daily; 0.3%	-	-		9.0	1.46

BalanCD CHO	-	-	-	-	1, 3, 5; 10%	7.1	1.30

The time [d] for feed addition and the feed volume in % of the culture volume are indicated. Feed start for the culture in BalanCD CHO was day 1, all other cultures were fed from day 3 on. Values for peak cell concentration and harvest titer are mean values of triplicate experiments.

• Fed-batch cultures were run in triplicates. The residual glucose concentration was maintained above 3 g/L by addition of glucose concentrate

• Analytics: cell concentration, viability, selected metabolites, product concentration, amino acid concentrations

## Results and discussion

**Figure 1:**
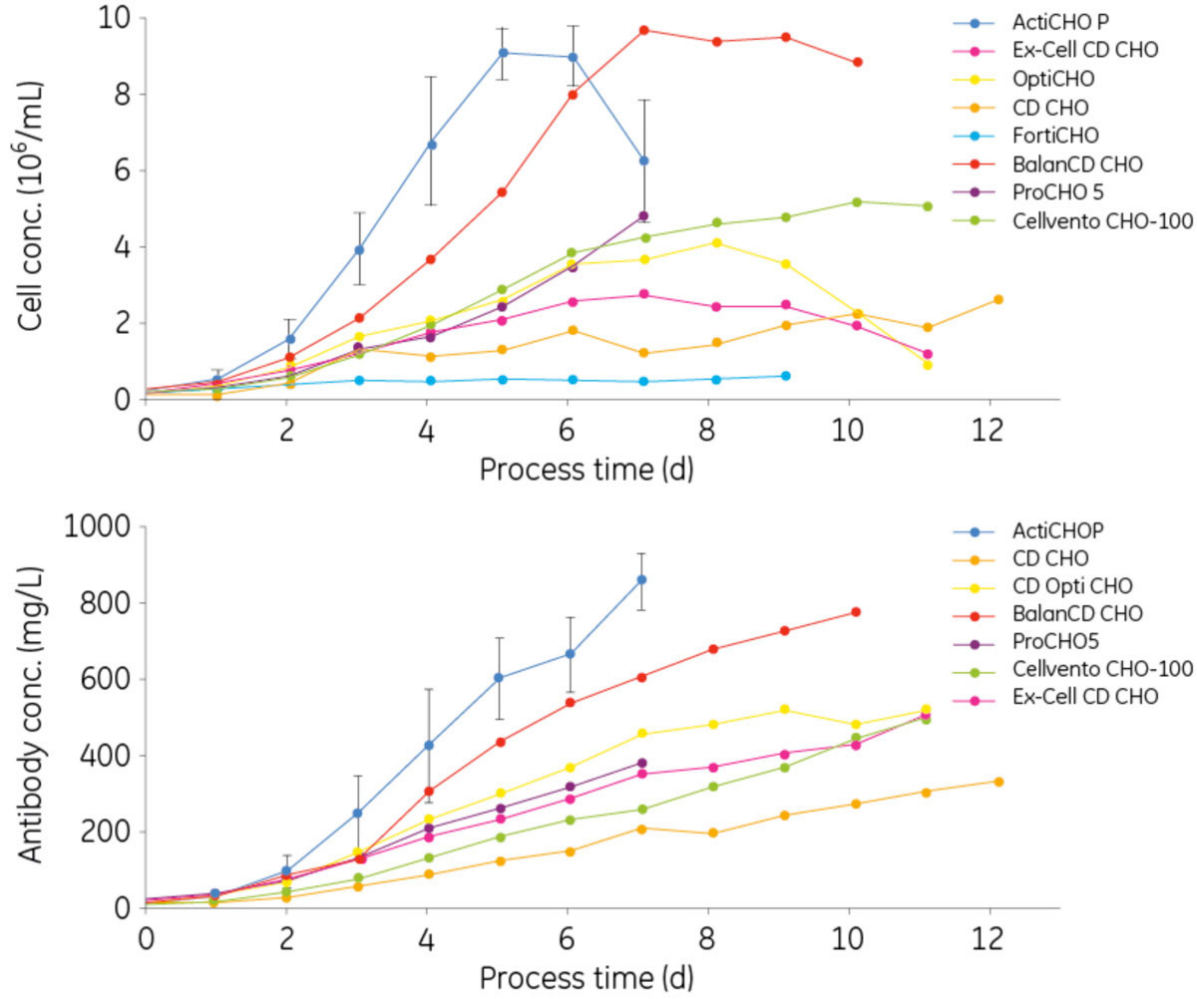
**Cell concentrations (upper panel) and product concentrations (lower panel) obtained in batch experiments with different commercially available CHO cell culture media**. Titers in CD FortiCHO were not determined due to low cell concentrations. Error bars are one standard deviation.

In batch cultures the highest cell concentrations were obtained in ActiCHO P and BalanCD as shown in Figure 1. In ActiCHO P the cells initially grew with a slightly higher specific growth rate (data not shown) and therefore the maximum cell concentration was reached 3 days earlier than in BalanCD. In ProCHO 5, Cellvento CHO-100 and CD OptiCHO, cell concentrations of 4 × 10^6 ^to 5 × 10^6 ^cells/mL were reached. Although initially the growth was similar in all three media, the culture in ProCHO 5 was terminated on day 7 due to a viability below 60%. In the other two media the batch lasted for four days longer. In Ex-Cell CD CHO cells grew to 2.6 × 10^6 ^cells/mL which was about 30% of the cell concentration reached in ActiCHO P. Finally in CD CHO and CD FortiCHO cells formed small aggregates and rather low concentrations of 2.5 × 10^6 ^and 6.0 × 10^5 ^cells/mL were obtained, respectively. Cell adaptation in CD FortiCHO during seven passages and addition of Anti-Clumping Agent (1:250) did not resolve the aggregation problem or improve cell growth (data not shown). The antibody production in the different cultures followed the same ranking as the cell growth (Figure 1). The highest titers were achieved in ActiCHO P and BalanCD CHO. In CD OptiCHO, Ex-Cell CD CHO and Cellvento CHO-100 product concentrations of about 500 mg/L were reached. The lowest titers were generated in ProCHO 5 and CD CHO with 380 mg/L and 330 mg/L, respectively. Fed-batch cultivations were then run in selected basal media with the respective feeds according to table 1. Again there was a strong correlation between cell concentration and antibody production. The highest cell and product concentrations were obtained in ActiCHO P (Table 1). Compared with the previous batch cultures, the cell concentrations were more than doubled and due to the extended process duration the titer was increased more than 6 fold, as shown in table 1. Feeding cultures in ActiCHO P with Feed A and B alone or additionally with FunctionMAX, altered the process only marginally. Supplementing the fed-batch only with ActiCHO Feeds A&B resulted in slightly higher cell concentrations and the process duration was reduced by 2 days (data not shown). A fed-batch culture in BalanCD medium and Feed 1 reached only 80% of the cell concentration achieved during the previous batch culture, however, feeding extended the process by five days and increased the antibody concentration by 60% compared with the previous batch culture to a final titer of 1.3 g/L (Table 1). Fed-batch cultures in CD OptiCHO achieved about 40% of the cell concentrations in ActiCHO P. Similar cell concentrations were reached when feeding cultures in CD OptiCHO with ActiCHO feeds A and B or EfficientFeed A, independent if the feed was added during 7 or 9 days or if additional feeding with FunctionMAX was performed (Table 1). However, the feeding had an impact on the product concentration. The lowest one was obtained when feeding cultures in CD OptiCHO with EfficientFeed A only. Further supplementation with FunctionMAX or feeding with ActiCHO Feed A&B substantially increased the product concentration (Table 1).

## Conclusions

• Batch cultivation in the different media resulted in peak cell concentrations from 2.5 × 10^6 ^to 9.0 × 10^6 ^cells/mL and a corresponding antibody titer from 220 to 860 mg/L. ActiCHO P and BalanCD CHO performed best in these cultures.

• Fed-batch cultivations substantially improved cell and product concentration. Feeding cultures in CD OptiCHO with EfficientFeed A and FunctionMAX or with Feed A and Feed B resulted in similar antibody concentrations and roughly doubled the antibody production compared to feeding with EfficientFeed A only.

• The highest titer was achieved in ActiCHO P in combination with Feed A and Feed B. In this medium a 6.3-fold improvement, compared with the previous batch cultivation, was observed. Further addition of FunctionMAX to these cultures did not significantly improve the antibody production.
